# Mining online activity data to understand food consumption behavior: A case of Asian fish sauce among Japanese consumers

**DOI:** 10.1002/fsn3.622

**Published:** 2018-03-09

**Authors:** Mitsutoshi Nakano, Hiroaki Sato, Toshihiro Watanabe, Katsumi Takano, Yoshimasa Sagane

**Affiliations:** ^1^ Department of Food and Cosmetic Science Faculty of Bioindustry Tokyo University of Agriculture Abashiri Japan; ^2^ The Organization for the Promotion of International Relationship Tokyo Japan; ^3^ Department of Applied Biology and Chemistry Faculty of Applied Bioscience Tokyo University of Agriculture Tokyo Japan

**Keywords:** consumer preference, consumption behavior, fish sauce, Japan, Japanese food, online recipes, online search, Thai food, Vietnamese food

## Abstract

Internet search engines and online recipe repositories have become increasingly popular resources among households for recipes and meal planning. Meanwhile, fish sauce's distinct flavor makes it a popular condiment in Southeast Asian countries. Although fish sauce is used as a condiment for traditional cuisine in Japan, it is not popular for general household use. To understand the consumption behavior regarding fish sauce in Japanese households, we analyzed search trends for the words *nampla* (Thai fish sauce), *nuoc mam* (Vietnamese fish sauce), and *shottsuru* (Japanese fish sauce) using Google's search engine and the Japanese online recipe site Cookpad. The results clearly indicate *nampla*'s rising popularity due to the rapid spread of Thai cuisine and an annually increasing traditional consumption of Japanese fish sauce. These results provide insights into the household demand for fish sauce.

## 1 INTRODUCTION

Consumers choose food based on many factors, such as flavor, appearance, nutritional value, function, and the package's label (Borgmeier & Wstenhoefer, 2009). Further, familiarity with the food is an important factor in consumer acceptance (Verneau, Caracciolo, Coppola, & Lombardi, 2013). Food researchers and developers analyze consumers' preferences and acceptance by conducting consumer surveys and interviews (Honkanen & Frewer, 2009; Kearney, Kearney, Dunne, & Gibney, 2000; Milošević, Žeželj, Gorton, & Barjolle, 2012); they can then construct new product concepts and produce pilot‐scale products based on this research. This traditional approach to product development requires many respondents for questionnaires, interviews, and sensory evaluations. The quality of the questionnaire and interview analyses primarily depends on the prepared questions as well as the interviewers' capabilities (Bastian, Eggett, & Jefferies, 2015).

Currently, information technology has enabled the accumulation of large amounts of data from social Web sites and the simple extraction of such data using search engines. Economists (Carrière‐Swallow & Labbé, 2013; Choi & Varian, 2012; Vosen & Schmidt, 2011), policymakers (Ripberger, 2011), epidemiologists (Carneiro & Mylonakis, 2009; Seifter, Schwarzwalder, Geis, & Aucott, 2010), and biologists (Mccallum & Bury, 2013; Proulx, Massicotte, & Pépino, 2013) increasingly use accumulated online data to understand markets, public opinion trends, and the spread of human infectious diseases, and to observe creatures' behaviors. Recently, in addition to Web sites that provide general information, online recipe sites—such as Cookpad (https://www.cookpad.com) and Allrecipes (http://www.allrecipes.com)-have become popular among households to find recipes shared by users and plan meals.

Meanwhile, fish sauce is a brown dressing widely consumed in most Southeast Asian countries and is referred to by different names across countries: *nampla* in Thailand, *nuoc mam* in Vietnam, and *shottsuru* in Japan (Akita Prefecture in particular). Generally, fish sauce is produced by fermenting fish material and salt (Fukami, Funatsu, Kawasaki, & Watabe, 2004), and microorganisms are often used to catalyze fermentation. During this fermentation process, the proteins in the fish material are hydrolyzed into amino acids and peptides, both by endogenous and by microorganism proteases, which results in the fish sauce's distinctive taste (Lopetcharat, Choi, Park, & Daeschel, 2001; Taira, Funatsu, Satomi, Takano, & Abe, 2007). The fermentation process also produces volatile compounds responsible for its characteristic odor (Yongsawatdigul, Rodtong, & Raksakulthai, 2007; Zheng et al., 2017).

Soy sauce and miso are fermented foods made from soybeans and are widely consumed as condiments in Japan. However, it is difficult to acquire soy sauce and miso in coastal areas due to a lack of soybeans. Therefore, fish are used to produce a substitute for soy sauce; fish sauce is primarily homemade, resulting in a unique fish sauce‐related food culture in limited areas of Japan. Nonetheless, homemade fish sauce production declined due to the emergence of industrial production and subsequent commercial availability of soy sauce and miso, caused by a drastic change in Japanese society after the Meiji era (Ishige, 1986). Thereafter, fish sauce was only produced in limited areas (i.e., *shottsuru* in Akita Prefecture and *ishiru* in Ishikawa Prefecture). Our recent investigation indicates that the Japanese people have gradually lost interest in fish sauce produced in Japan (Nakano et al., 2017). Despite waning consumer demand, fish sauce production has gained attention, and many local fish sauce products are produced to revitalize local communities, which have an advantageous, abundant supply of inedible fish material at a low market value that can be used for fish sauce production. This indicates a substantial gap between consumer demand and supply.

In this study, we use Google Trends, which enables the plotting of Google search queries related to a particular topic, to analyze fish sauce consumption behaviors in Japan. Additionally, we employ Tabemiru, a tool provided by the online recipe repository Cookpad that enables an analysis of recipe use frequencies, to examine households' fish sauce consumption. The combined data from Google Trends and Tabemiru represent Japan's household fish sauce consumption behavior.

## 2 MATERIALS AND METHODS

### 2.1 Data collection from Google Trends

Temporal or regional trends in web searches for the terms of concern in this study were downloaded from Google Trends (https://trends.google.com/trends/). This public web facility of Alphabet Inc. (Mountain View, CA, USA) indicates how often people search for a term relative to the total number of searches, in various countries and languages. This study's data were collected by setting the location parameter to “Japan” and the time parameter to “2004‐present.”

### 2.2 Data collection from Tabemiru on Cookpad

Cookpad, Japan's largest recipe site (http://www.cookpad.com), allows visitors to upload and search for original recipes. Tabemiru is an analysis tool provided by Cookpad Inc. (Tokyo, Japan) that allows visitors to examine how often Cookpad visitors use recipes, including relevant search terms. The frequency is expressed as the cook index (CI), according to which Tabemiru considers a recipe that has been uninterruptedly viewed for more than 10 min as “prepared by the site visitor.” The CI is calculated using the following formula: the number of times a recipe, including relevant search terms, was used for cooking/total number of recipes that were used ×1,000.

### 2.3 Statistical analysis

The correlation between Google Trends‐based search volumes and household consumption behavior, as represented by the CI in Tabemiru, was assessed with scatterplots and regression statistics using both the coefficient of determination and Pearson's correlation coefficient. Additionally, the correlations between the search volumes for the terms for fish sauce and the dishes' names were similarly assessed. All statistical analyses were performed using Microsoft Excel (version 15.31; Redmond, WA, USA).

## 3 RESULTS

### 3.1 Google search trends for fish sauce among Japanese consumers

**Figure 1:**
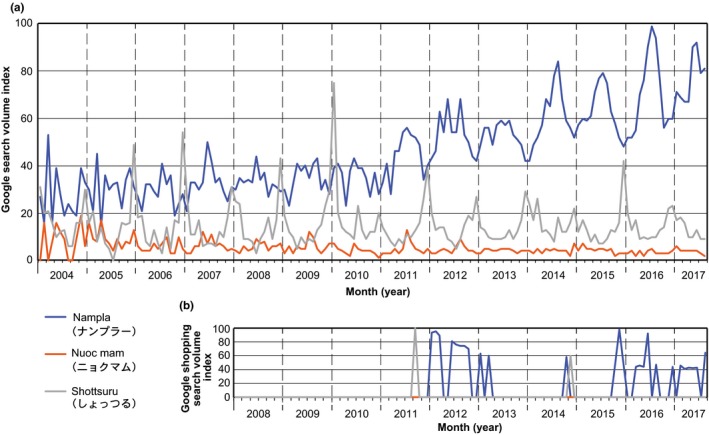
(a) Interest in *nampla*,* nuoc mam,* and *shottsuru* and (b) in buying those fish sauce types. This information is represented by the Google search volumes for the query terms ナンプラー (*nampla*), ニョクマム (*nuoc mam*), and しょっつる (*shottsuru*), from 2004 to 2017

We entered the set of terms “ナンプラー”(*nampla*; Thai fish sauce), “ニョクマム”(*nuoc mam*; Vietnamese fish sauce), and “しょっつる” (*shottsuru*; Japanese fish sauce) on the Google Trends homepage in August 2017 to access the search trends for these three keywords. Figure 1a illustrates a Google Trends graph of the search frequencies for the three terms, from January 2004 to August 2017. Among the three terms, searches for *nampla* had the most traffic, or an average of nine times more than the *nuoc mam* searches, and three times more than the *shottsuru* searches. It is noteworthy that search trends for *nampla* peak in the summer and reach its minimum in the winter, which are remarkable after 2011. Conversely, the search trend for *shottsuru* demonstrates a sharp peak in December. Such seasonal search trends were not observed for *nuoc mam*. The search trends for the three terms for fish sauce on Google Shopping were also analyzed, as Figure 1b reveals. This indicates consumers' willingness to buy and a stronger interest than that of consumers who search using only Google's homepage. Searches for *nampla* occurred every year after 2012, while searches for *shottsuru* occurred only in the winters of 2011 and 2014. *Nuoc mam* searches were not observed for any year.

**Figure 2:**
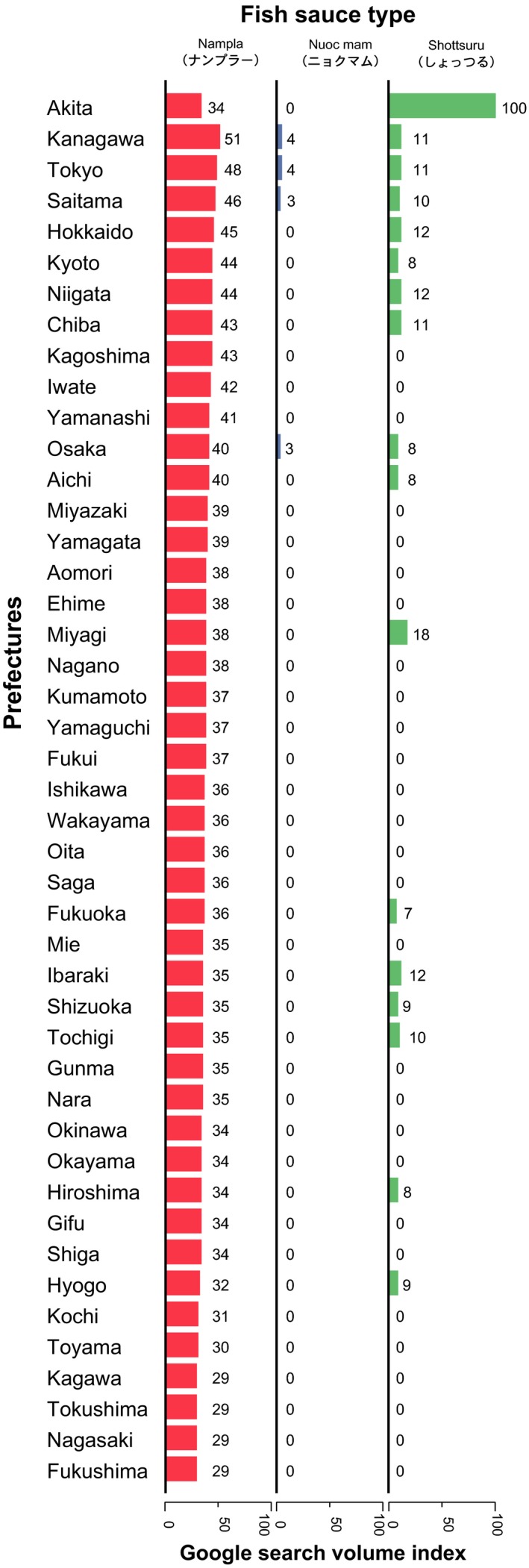
Interest in *nampla*,* nuoc mam*, and *shottsuru* by prefecture. Each bar indicates the Google search volume index

Google Trends also lists the prefectures with the highest search traffic for a given term. As Figure 2 demonstrates, 17 of 47 prefectures were listed for *shottsuru* searches, with Akita Prefecture indicating remarkably high search traffic. Alternatively, all prefectures in Japan widely searched for *nampla*. Searches for *nuoc mam* only occurred in metropolitan areas with large populations, such as in Kanagawa, Tokyo, Saitama, and Osaka prefectures.

### 3.2 Household consumption behavior for fish sauces in Japan

**Figure 3:**
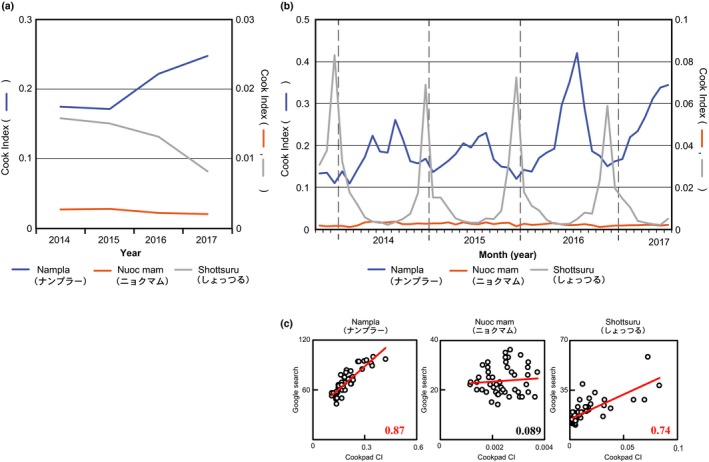
(a) Annual and (b) monthly trends in the use of Cookpad recipes including each fish sauce type. This information is represented by the cook index (CI). The scatterplots and regression statistics indicate a strong positive and linear relationship between the Google search trends and CIs for *nampla* and *shottsuru*, (c) but not for *nuoc mam*

We clarified the household consumption behavior for the three fish sauce types in Japan by analyzing their frequency from 2014 to 2017 in recipes that include *nampla*,* nuoc mam*, and *shottsuru* on Japan's largest online recipe repository, Cookpad. This was accomplished using Cookpad Inc.'s analysis tool, Tabemiru, which expressed this frequency as its cooking index (CI). As Figure 3a reveals, the frequency of the use of recipes that include *nampla* was 10 times higher than that of *shottsuru*, and 80 times higher than that for *nuoc mam*. The frequency of the use of recipes that include *nampla* increased every year after 2015, while that of *shottsuru* decreased every year since 2014.

Figure 3b compares the monthly trends regarding the frequency of use for recipes that include the three fish sauce types. Similar to Google's search trends, the frequency of use of recipes that include *nampla* peaked in the summer, while those including *shottsuru* sharply peaked in winter. As the monthly Google search trends and the recipes' frequency of use exhibit similar behaviors, Pearson's correlation coefficient was used to analyze the correlation between these two datasets, as noted in Figure 3c. The results reveal highly positive correlations for *nampla* and *shottsuru* in the Google search frequency and recipes' frequency of use as represented by the CI. The Pearson's correlation coefficient was 0.87 for *nampla*; consequently, a high correlation exists between people's interest and household consumption for both *nampla* and *shottsuru*.

**Figure 4:**
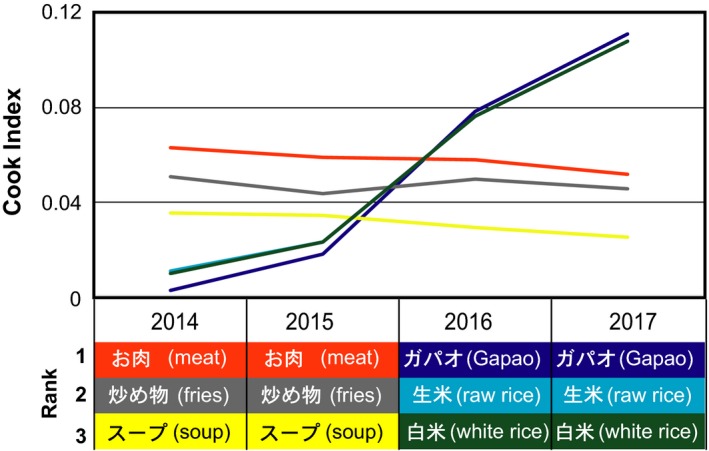
Annual data on foods that use *nampla* from a combination search on Tabemiru on Cookpad. The bottom panel indicates the top three retrieved terms for dishes that use *nampla* fish sauce

**Figure 5:**
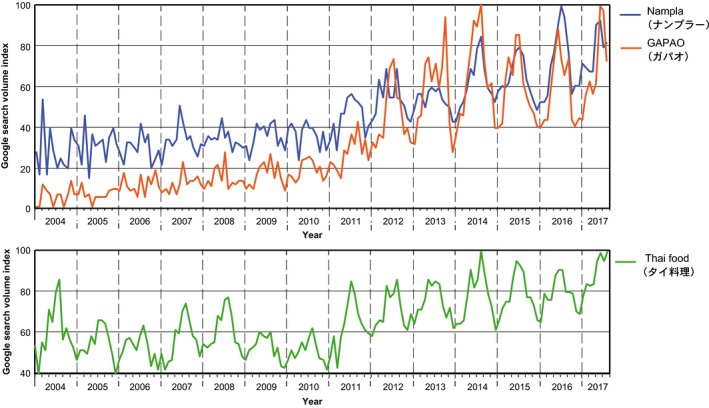
Interest in (a) *nampla* and *gapao* and (b) Thai food. This information is represented by the Google search volumes for the query terms ナンプラー (*nampla*), ガパオ(*gapao*), and タイ料理 (Thai food), from 2004 to 2017

Next, we analyzed foods that used *nampla*,* nuoc mam*, and *shottsuru* in the recipes on Cookpad using Tabemiru. As Table 1 indicates, *shottsuru* is almost always used as a condiment in *shottsuru nabe*, a Japanese stew with *konbu* (kelp) and *hata‐hata* (Japanese sandfish). The stew is a traditional dish in Akita Prefecture (Ishige, 1986). The data also indicate that *nuoc mam* is used as a dipping sauce for various foods or a condiment for fried chicken or other meat. Alternatively, the data also indicate a drastic change since 2016 in *nampla*'s household use. Figure 4 indicates the CI for each food that used *nampla*; the constant CI scores for meat, fries, and soup throughout 2014 to 2017 imply that *nampla* was used as a condiment for fried meat, fries, and soup. Further, the data demonstrate a rapidly increasing interest in the search terms “*gapao*” and “*rice*,” referring to a new Thai dish called *gapao* rice that became popular in Japan around 2015 to 2016. The upper panel in Figure 5 indicates a Google Trends graph of the search frequencies for ナンプラー (*nampla*) and ガパオ (*gapao*) from January 2004 to August 2017. This panel implies that the trends in *nampla*'s popularity are strongly linked to those of *gapao*, and these terms have increased in popularity since 2012. Additionally, the search frequency for the termタイ料理 (Thai foods) has increased since 2012. These results indicate that Thai cuisine has gradually become popular in Japanese society, and especially with the increasing popularity of *gapao* rice, which has been consumed in households as well as restaurants specializing in Thai cuisine.

**Table 1:** Top 3 retrieved terms for dishes that use each fish sauce type

Rank	*Nampla*(ナンプラー)				*Nuoc mam*(ニョクマム)				*Shottsuru*(しょっつる)			
	2014	2015	2016	2017	2014	2015	2016	2017	2014	2015	2016	2017
1	お肉 (Meat)	お肉 (Meat)	ガパオ^a^ (*Gapao*)	ガパオ (*Gapao*)	タレ^b^ (*Tare*)	炒め物 (Fries)	お肉 (Meat)	タレ (*Tare*)	鍋^c^ (*Nabe*)	鍋 (*Nabe*)	鍋 (*Nabe*)	鍋 (*Nabe*)
2	炒め物 (Fries)	炒め物 (Fries)	生米 (Raw rice)	生米 (Raw rice)	炒め物 (Fries)	お肉 (Meat)	炒め物 (Fries)	お肉 (Meat)	コンブ^d^ (*Konbu*)	はたはた (*Hata‐hata*)	はたはた (*Hata‐hata*)	はたはた (*Hata‐hata*)
3	スープ (Soup)	スープ (Soup)	白米 (White rice)	白米 (White rice)	お肉 (Meat)	鶏 (Chicken)	鶏 (Chicken)	炒め物 (Fries)	はたはた^e^ (*Hata‐hata*)	コンブ (*Konbu*)	コンブ (*Konbu*)	コンブ (*Konbu*)

a ガパオ (*Gapao*): Japanese pronunciation for กะเพรา, the Thai name for holy basil (*Ocimum tenuiflorum*), used in Thai cuisine. *Gapao* is a dish made of minced meat, fried with holy basil or sweet basil, and served with rice.

b タレ (*Tare*): A dipping sauce for cooked food, such as *yaki‐niku* (Korean‐style barbecue) and *gyoza* (a dish that evolved from Chinese dumplings).

c 鍋 (*Nabe*): Originally interpreted as a pan for boiling or stewing, the term also refers to food boiled in a *nabe*.

d コンブ (*Konbu*): Kelp used to make *dashi*, a broth in various Japanese dishes.

e はたはた (*Hata‐hata*): Japanese sandfish (*Arctoscopus japonicas*).

### 3.3 *Nampla*'s increasing popularity as a condiment in Southeast Asian cuisine

**Figure 6:**
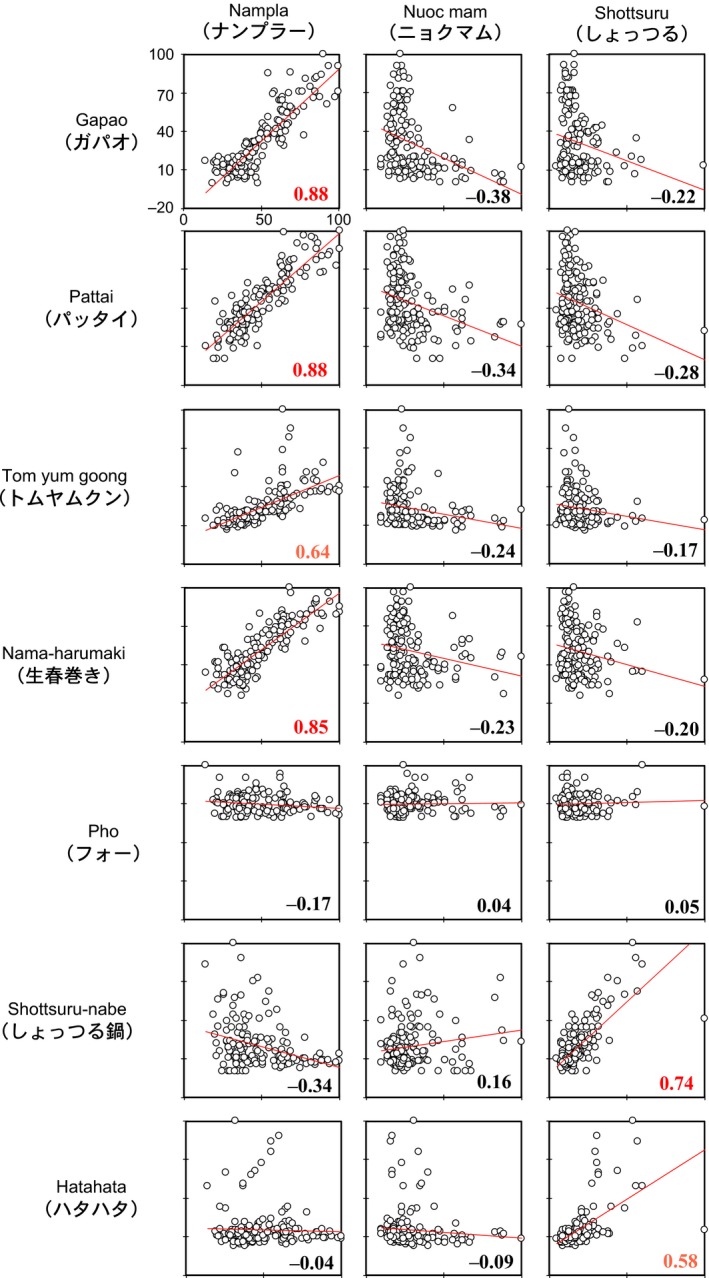
Monthly Google search volume scatterplots per fish sauce type (*x*‐axis) and dish (*y*‐axis). The correlations were assessed with regression lines and Pearson's correlation cohesion

As Figure 6 illustrates, *nampla*'s search frequency significantly relates to those for *gapao*,* pattai*,* tom yum goong*, and *nama‐harumaki*, which suggests that the people's interest in *nampla* relates to these dishes. Indeed, some Cookpad recipes for these dishes list *nampla* as a condiment. It is also noteworthy that *nama‐harumaki* originated in Vietnam and uses *nampla* in place of *nuoc mam*. The search frequency trends for *nuoc mam* did not indicate relationships to any of the dish names analyzed in this study, including the Vietnamese dishes *nama‐harumaki* and *pho*. The search trends for *shottsuru* relate to both *shottsuru nabe* and *hata‐hata*.

**Figure 7:**
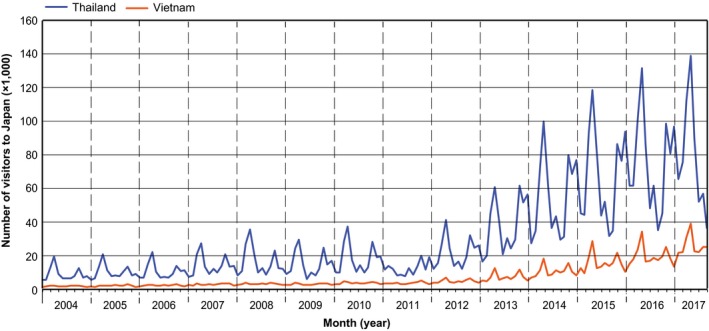
Monthly data on visitors from Thailand and Vietnam to Japan. The line graph was prepared based on data from the Japan National Tourism Organization (https://www.jnto.go.jp)

One reason for Thai cuisine's rapidly increasing popularity in Japan is the increasing number of visitors from Thailand. Figure 7 indicates that the number of visitors from Thailand to Japan has significantly increased since 2013. Moreover, the Japanese government permitted people from Thailand to enter the country without a visa in July 2013. This increase in visitors from Thailand may have promoted a cultural exchange, including the nations' cuisines.

## 4 DISCUSSION

Fish sauce has been popular among Japanese households as a substitute for soy sauce, which is a condiment for traditional dishes, or as a dipping sauce (*tare*) for raw fish (*sashimi*) (Ishige, 1986). However, drastic changes in Japanese society have led to a decline in fish sauce consumption. Nevertheless, the production of a wide variety of fish sauces has continued throughout Japan, as some local communities have an abundant supply of fish and inedible fish material at a low market value, which can be used to manufacture the product. However, strategies to promote sales and product development are necessary for the industry to succeed. This study performed data mining on Internet activities (searches) related to fish sauce consumption among the Japanese.

The data mined from Google Trends indicate that fish sauce produced in Japan, represented by *shottsuru*, has a very limited purpose as a condiment for traditional *nabe* (Ishige, 1986; Nakano et al., 2017). Thus, there is currently an excess supply of fish sauce relative to consumer demand. Conversely, *nampla* has become widely popular in Japan due to Thai cuisine's rapidly increasing popularity in recent years, as represented by *gapao* rice. The data mined from Cookpad indicate that Thai cuisine has now spread to households, beyond the restaurants that serve such cuisine. These findings may have significant implications for the Japanese fish sauce industry. On the one hand, *gapao* rice's increasing popularity in Japanese society has led to an increased consumption of *nampla*. On the other hand, no dish has led to an increased consumption of Japanese fish sauce, and therefore, it is necessary to develop dishes that use this fish sauce. Additionally, suppliers must explain the benefits of unfamiliar foods to consumers or their households. For instance, fish sauce can be incredibly nutritious because of its essential amino acids (Gildberg, 2004). Additionally, fish sauce promotes various biological activities, including angiotensin I‐converting enzyme inhibitory activity and the stimulation of insulin production (Ichimura, Hu, Aita, & Maruyama, 2003). The distribution of such useful information to consumers would be part of a sales strategy beneficial to the Japanese fish sauce industry.

The data mined from Google Trends and Tabemiru indicate the unique seasonal differences in consuming *nampla* and *shottsuru*, with peaks in the summer and winter, respectively. Japanese consumers tend to make spicy foods, such as ethnic foods, in the summer to prevent a loss of appetite. Recently, the cultural exchanges between Japan and Thailand have increased Thai foods' popularity as ethnic foods. Moreover, *nabe* cuisine is popular in the winter season. These reasons justify the seasonal trends demonstrated in Google searches for *shottsuru* as well as its usage on the Cookpad Web site.

Consumer research and sales data collection are needed for businesses' marketing research to understand consumers' preferences. This study indicates that an analysis of Internet data—using search trends from an Internet search engine and an online recipe Web site—provides a method to observe consumer preferences that complement existing methods.

## CONFLICT OF INTEREST

None declared.
